# PELI1 in human cancers: a pan-cancer exploration of its molecular function, clinical significance, and immunomodulatory roles

**DOI:** 10.3389/fimmu.2025.1682086

**Published:** 2026-01-05

**Authors:** Yan Xu, Jiale Zhou, Xiaoran Chen, Xiaoqing Dong, Bing Chen

**Affiliations:** 1 Department of Hematology, Nanjing Drum Tower Hospital, Affiliated Hospital of Medical School, Nanjing University, Nanjing, Jiangsu, China; 2 Department of Hematology, Nanjing Drum Tower Hospital Clinical College of Nanjing University of Chinese Medicine, Nanjing, Jiangsu, China

**Keywords:** PELI1, pan-cancer, prognosis, diagnosis, immune infiltration, biological function

## Abstract

**Introduction:**

The E3 ubiquitin ligase Pellino1 (PELI1) is ubiquitously expressed in human tissues and primarily modulates inflammatory and immune responses; however, its pan-cancer biological significance remains poorly characterized.

**Methods:**

We employed a combination of R software and online bioinformatics platforms—including UALCAN, HPA, GEPIA2, cBioPortal, STRING, TISIDB, SRAMP, LinkedOmics, and Sangerbox—to systematically characterize PELI1 in human tumors. Our analyses encompassed its abnormal expression, genetic alterations, prognostic and diagnostic relevance, and epigenetic regulation. Focusing on liver hepatocellular carcinoma (LIHC), we further explored the oncogenic functions of PELI1, its associated signaling pathways, and immunomodulatory roles. Key bioinformatic predictions were subsequently validated through *in vitro* experiments using LIHC cell lines, including functional assays (proliferation, apoptosis, and cell cycle) and signaling pathway analyses.

**Results:**

PELI1 was frequently overexpressed across multiple tumor types and mainly correlated with poor patient prognosis. The highest frequency of PELI1 alterations was observed in patients with diffuse large B-cell lymphoma, primarily in the form of copy number amplification, while R145Q/W represented the most recurrent mutation sites. PELI1 may drives cancer progression through epigenetic regulation involving DNA methylation and N6-methyladenosine (m6A) RNA modifications. PELI1 could potentially serve as a novel biomarker for diagnosing different cancer types. In LIHC specifically, PELI1 expression was significantly elevated compared to adjacent non-tumor tissues. Functional studies revealed that PELI1 modulated cell apoptosis, cell cycle progression, immune responses and the MAPK-ERK pathway in LIHC. Furthermore, PELI1 may regulate immune checkpoints to influence the immune responses. PELI1 expression was positively correlated with the IC50 of hepatocellular carcinoma investigational drug–CFI-402257. Experimental knockdown of PELI1 in LIHC cell lines suppressed proliferation, promoted apoptosis and induced G1-phase cell cycle arrest via MAPK-ERK pathway.

**Conclusions:**

This pan-cancer study revealed the diagnostic, prognostic and immunomodulatory potential, along with the biological function and mechanism of PELI1, supporting its role as a promising therapeutic target in LIHC.

## Introduction

Cancer represents a major global public health challenge, with its escalating incidence posing a serious threat to human health and emerging as one of the leading causes of mortality worldwide (1). Despite significant advancements in medicine science and improvements in cancer diagnosis and treatment over recent years, tumor underdiagnosis and misdiagnosis remain prevalent, while the overall 5-year survival rate across cancer types continues to be suboptimal (2). This underscores the urgent need for innovative diagnostic and therapeutic targets and approaches. Notably, studies restricted to individual cancer types have constrained our ability to gain a holistic understanding of cancer genetics and underlying mechanisms. Characterizing the molecular similarities and discrepancies across diverse tumor types is therefore crucial for elucidating the fundamental dynamics of tumorigenesis and informing precision diagnosis, prognosis, and therapeutic strategies. Pan-cancer analysis, an approach that interrogates molecular aberrations across multiple cancer types, integrates and summarizes cellular features across different lineages to uncover dysregulated key biological processes (3). Elucidating pan-cancer gene signatures has thus become an indispensable tool for unraveling the mechanisms of cancer genetics.

E3 ubiquitin ligases serve as central regulators of the ubiquitin-proteasome system through their ability to specifically recognize substrate proteins and mediate their ubiquitination, thereby governing protein stability, subcellular localization, and functional activity (4). This regulatory axis orchestrates critical biological processes including cell proliferation, apoptosis, DNA damage repair, and immune responses (5). Within the E3 ubiquitin ligase family, Pellino1 (PELI1) has initially gained prominence through its regulatory roles in inflammation and immune signaling cascades mediated by Toll-like receptors and interleukin-1 receptors. Emerging evidence has highlighted aberrant PELI1 expression across a spectrum of malignant neoplasms (e.g., lung cancer, breast cancer, and lymphoma), where it regulates tumor cell survival, invasion, and metastasis by modulating key signaling pathways such as PI3K-AKT, NF-κB and B-cell signaling pathways (6).

However, a comprehensive pan-cancer characterization of PELI1 remains unexplored. A systematic investigation of PELI1—including its expression profiles, clinical significance, immune correlations, and underlying molecular mechanisms—could provide novel mechanistic insights into tumorigenesis and facilitate the development of innovative cancer therapies. To address this gap, we leveraged multi-omics data from The Cancer Genome Atlas (TCGA) and other publicly available repositories to conduct an extensive pan-cancer analysis of PELI1. Our investigations encompassed expression profiles, genetic mutation analysis, DNA methylation patterns, N6-methyladenosine (m6A) modification status, diagnostic utility assessment, prognostic outcomes, immune correlation analysis and drug efficacy analysis. Furthermore, by integrating bioinformatics predictions with experimental validation, we elucidated the expression patterns, biological functions and functional pathways of PELI1 in liver hepatocellular carcinoma (LIHC). Collectively, our findings reveal that PELI1 exerts multifaceted roles across pan-cancer, influencing diagnostic accuracy, prognostic outcomes, and the tumor immune microenvironment. These results underscore PELI1 as a promising therapeutic target with broad relevance to LIHC.

## Materials and methods

### Expression analysis of PELI1

PELI1 gene expression levels were compared between normal and tumor tissues across various tumor types present in The Cancer Genome Atlas (TCGA, https://portal.gdc.cancer.gov/). The statistical differentiation was assessed by Wilcoxon signed-rank test. PELI1 expression at the protein levels were analyzed using the Clinical Proteomic Tumor Analysis Consortium (CPTAC) database. Differential PELI1 expression across pathological stages was visualized using violin plots, generated through the University of Alabama at Birmingham Cancer (UALCAN) online tool (7). Additionally, PELI1 expression profiles in cancer cell lines and tumor tissues were extracted from the Human Protein Atlas (HPA, https://www.proteinatlas.org) (8). The hepatocellular carcinoma (HCC) tumor and adjacent tissues datasets were obtained from hepatocellular carcinoma database (HCCDB) (http://lifeome.net/database/hccdb/home.html) (9) for PELI1 expression validation in LIHC. Additionally, the RNA-seq data of GSE64041, GSE84402, GSE76427 were downloaded from Gene Expression Omnibus (GEO, https://www.ncbi.nlm.nih.gov/) (10). Furthermore, the differential expression of PELI1 in LIHC was simultaneously validated using the TNMplot database (11). Single-cell RNA sequencing analysis of hepatocellular carcinoma was conducted using Single Cell Portal (https://singlecell.broadinstitute.org/single_cell).

### Genetic alteration analysis

The cBioPortal database (https://www.cbioportal.org/) (12) was utilized to analyze PELI1 genetic alterations across tumors using data from the TCGA Pan-Cancer Atlas Studies. In the ‘Cancer Type Summary’ module, we quantified the mutation frequency and copy number variations (CNVs) of the PELI1 gene. Additionally, the ‘Mutations’ module was employed to generate a comprehensive mutation site profile of PELI1. To assess the clinical relevance of these alterations, Kaplan-Meier survival analyses were performed for specific TCGA cancer types using the ‘Comparison/Survival’ module, comparing cohorts with and without PELI1 genetic alterations.

### DNA methylation and m6A modification analysis

The gene promoter methylation levels of PELI1 between normal and tumor groups were analyzed from TCGA database. MethSurv (https://biit.cs.ut.ee/methsurv/) was used to visualize DNA methylation of PELI1 in tumors. Sangerbox was used to conduct the correlation analysis between PELI1 and 21 m6A regulators in different cancer types based on TCGA database. The prediction of m6A modification sites within PELI1 was conducted using the sequence-based RNA adenosine methylation site predictor (SRAMP) web tool (http://www.cuilab.cn/sramp/) (13).

### Survival analysis and diagnostic value of PELI1

The overall survival (OS) and disease-free survival (DFS) of PELI1 across all TCGA cohorts were evaluated using the ‘Survival Analysis’ module in GEPIA2 (14). Survival maps and Kaplan-Meier plots were generated to assess the prognostic significance of PELI1 expression. For the survival map, we systematically examined the association between PELI1 expression and patient outcomes across multiple cancer types, with statistical significance determined by the Mantel–Cox test. In the Kaplan-Meier analysis, cohorts were stratified into high- and low-PELI1 expression groups based on median expression values. Additionally, receiver operating characteristic (ROC) curves were constructed for selected tumors to further evaluate the prognostic value of PELI1.

### Functional enrichment analysis and PPI network analysis

A PPI network of the 30 PELI1 binding genes was created from the STRING database (15). Additionally, the GEPIA2 database was used to obtain the 100 genes most closely related to PELI1 in LIHC (14). Then, we performed Gene Ontology (GO) analysis and Kyoto Encyclopedia of Genes and Genomes (KEGG) analysis based on the PELI1 related genes to explore the potential functions of PELI1. Besides, we screened the genes that were significantly correlated with PELI1 expression in LIHC using LinkedOmics database (http://www.linkedomics.org/login.php) (16). And the correlation of results was tested by the Pearson correlation coefficient. The pathway and network analysis of these co-expressed genes were performed by the LinkInterpreter module, and gene set enrichment analysis (GSEA) tool was used to perform analyses of KEGG pathways and GO analysis. In LIHC, differentially expressed genes between the PELI1^high^ and PELI1^low^ groups were screened with an adjusted *P* < 0.05, |fold change| >2, and then analyzed for GO function and enriched KEGG pathways.

### Immune analysis

TISIDB was used to perform spearman correlation analysis of PELI1 expression and immune cell infiltration (17). Using the Kaplan-Meier plotter, the effects of PELI1 expression on the OS of patients with high or low levels of immune infiltration were also investigated. Sangerbox was used to conduct correlation analysis of PELI1 expression and immune checkpoint molecules. The association between PELI1 expression and immune cell infiltration was further assessed using ImmuCellAI, which is based on the single-sample gene set enrichment analysis (ssGSEA) algorithm (18). Additionally, the relationship between the gene set variation analysis (GSVA) enrichment score and immune infiltration was also examined.

### Analysis of drug treatment and PELI1 expression

We logged in CellMiner online database (https://discover.nci.nih.gov/cellminer/home.do) to get the correlation between pharmacological treatment efficacy and gene expression level.

### Cell culture and transfection

The human hepatocellular carcinoma (LIHC) cell line HepG2 was obtained from the Cell Bank of the Chinese Academy of Sciences. Cells were maintained in Dulbecco’s modified Eagle’s medium (DMEM; Keygen, Jiangsu, China) supplemented with 10% fetal bovine serum (FBS; Gibco) and 100 U/mL penicillin–streptomycin at 37 °C in a humidified 5% CO atmosphere. ShRNA oligonucleotides plasmids were designed and synthesized. The sequence of PELI1 shRNA (shPELI1) were 5’-CTCATTGTCTTAGGGTATAAT-3’, 5’-TTACAAGATGGCTCGTTAATT-3’. The retrovirus supernatant was packaged in HEK-293T cells by co-transfecting with object plasmid and packaging plasmid. Then viral supernatant was collected after transfection for 48 and 72 hours and purified with 0.45-μm filter. HepG2 cells were seeded in 12-well plates 24 hours prior to transfection. When cell confluence reached ~40%, the cells were transfected with viral supernatant in the presence of polybrene (8 μg/mL). After 6 hours, the medium was replaced with fresh complete medium. Transfected cells were harvested 24 hours post-transfection for subsequent experiments.

### Cell viability detection

Cell proliferation was assessed using the CCK-8 assay kit (Targetmol, Shanghai, China) according to the manufacturer’s instructions. Briefly, cells were seeded in 96-well plates at a density of 1×10^4^ cells/well and cultured under standard conditions (37°C, 5% CO_2_) for 24, 48, and 72 hours. At each time point, 10 μL of CCK-8 solution was added to each well, followed by incubation for 4 hours. The optical density (OD) was then measured at 450 nm using a microplate reader (Thermo Fisher Scientific, USA). Cell proliferation curves were generated by plotting OD values against time to compare proliferative capacity among different groups.

### Flow cytometric analysis

Cell cycle analysis. Cells were harvested, washed twice with ice-cold PBS, and fixed in 75% ethanol at -20°C overnight (minimum 6 hours). After fixation, cells were washed twice with PBS and treated with RNase A (10 μg/mL) at 37°C for 30 minutes to digest RNA. Cells were then stained with propidium iodide (PI; 20 μg/mL) in the dark. Cell cycle distribution was analyzed by flow cytometry, and data were processed using FlowJo software (version 10.8.1) to determine the percentage of cells in G0/G1, S, and G2/M phases.

Cell apoptosis was assessed using Annexin V/PI double staining (Keygentec, Nanjing, China) following the manufacturer’s protocol. Briefly, cells were harvested and washed twice with cold PBS, then resuspended in 1× binding buffer at a concentration of 1×10^6^ cells/mL. Cells were stained with 5 μL Annexin V and 10 μL PI (20 μg/mL) for 15 minutes at room temperature in the dark. Flow cytometry analysis was immediately performed, and the data were analyzed using FlowJo software (version 10.8.1) to distinguish apoptotic cell populations.

### mRNA extraction and quantitative RT-PCR

Total RNA was isolated using Trizol reagent (Invitrogen) following the manufacturer’s protocol. RNA purity and concentration were assessed using a NanoDrop spectrophotometer (Thermo Fisher Scientific). Subsequently, cDNA was synthesized from 1 µg of total RNA using a reverse transcription kit (TaKaRa, Japan). Quantitative real-time PCR (qRT-PCR) was performed using a SYBR Green PCR kit on an Applied Biosystems detection system (ABI), with gene-specific primers. The 18S ribosomal RNA (18S rRNA) served as the endogenous control for normalization. Gene expression was determined by the delta CT method (2-(ΔΔCt)). ΔCt = (Ct target gene - Ct housekeeping). The 18S primer sequences were F:5’-GCAATTATTCCCCATGAACG-3’, R:5’-GGCCTCACTAAACCATCCAA-3’. The PELI1 primer sequences were F:5’-TTGTGATGCATCCACGCAAT-3’, R:5’-ACAATGTTGCACCACAGAGG-3’.

### Immunoblotting

Cells were lysed in RIPA buffer (Beyotime) supplemented with a protease and phosphatase inhibitor cocktail (Beyotime) for 30 min on ice, followed by centrifugation at 12,000 × *g* for 15 min at 4°C to remove debris. The supernatant was collected, and protein concentration was determined using a bicinchoninic acid (BCA) assay kit (Beyotime) according to the manufacturer’s instructions. For immunoblotting, equal amounts of protein were separated by SDS-PAGE, transferred to PVDF membranes, and probed with specific primary antibodies.

### Statistical analysis

The statistical analyses of bioinformatic results in this study were presented by the online databases mentioned above. Survival analysis was performed using Kaplan-Meier curves and log-rank test. Experimental data are presented as mean ± standard deviation for the indicated number of separate experiments. Statistical analyses were performed with unpaired two-tailed Student’s t-test except where indicated otherwise using Prism (GraphPad) and R (version 4.3) software. *P* values < 0.05 were considered statistically significant. (*P < 0.05, **P < 0.01, ***P < 0.001, and ****P < 0.0001).

## Results

### The expression of PELI1 across tumors

**Figure 1:**
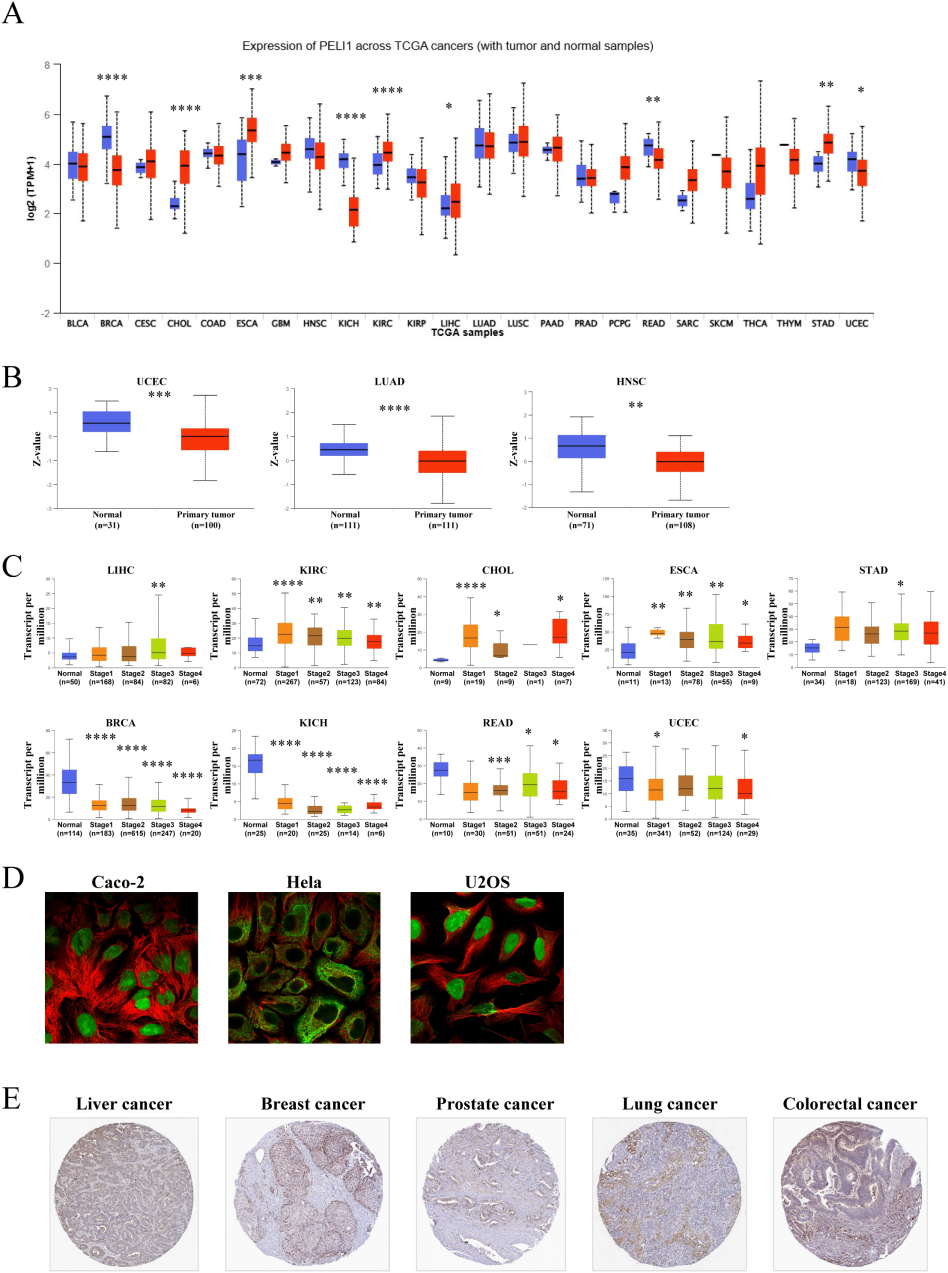
PELI1 expression in multiple tumors and major pathological stages**. (A)** Compared with paired normal samples, PELI1 gene expression levels in tumor samples from multiple cancer types were obtained based on The Cancer Genome Atlas (TCGA) data. **(B)** PELI1 protein expression levels between normal tissue and primary tissue of uterine corpus endometrial carcinoma (UCEC), lung adenocarcinoma (LUAD) and head and neck squamous carcinoma (HNSC) were analyzed according to data from the Clinical Proteomic Tumor Analysis Consortium (CPTAC) dataset. **(C)** The PELI1 gene expression levels were detected according to pathological stage in LIHC, BRCA, CHOL, ESCA, KIRC, KICH, READ, UCEC and STAD. Only statistically significant differences were shown. **(D)** PELI1 protein expression levels were detected by immunofluorescence. PELI1 was labeled with green fluorescence. Microtubules were labeled with red fluorescence. **(E)** Representative images of PELI1 expression in cancer tissue samples were shown. Staining: median. *P<0.05; **P<0.01; ***P<0.001; ****P<0.0001.

Dysregulated PELI1 expression in tumor tissues implies its involvement in oncogenesis. Analysis of normalized TCGA data revealed significant PELI1 upregulation across multiple malignancies, including cholangiocarcinoma (CHOL), esophageal carcinoma (ESCA), kidney renal clear cell carcinoma (KIRC), hepatocellular carcinoma (LIHC), and stomach adenocarcinoma (STAD) (**Figure 1A**, **Supplementary Table S1**). In contrast, protein-level data demonstrated PELI1 downregulation in uterine corpus endometrial carcinoma (UCEC), lung adenocarcinoma (LUAD), and head and neck squamous cell carcinoma (HNSC) (**Figure 1B**). Pathological stage progression correlated with increasing PELI1 expression in LIHC, KIRC, CHOL, ESCA, and STAD, but decreasing levels in breast invasive carcinoma (BRCA), kidney chromophobe (KICH), rectal adenocarcinoma (READ), and UCEC (**Figure 1C**). Immunofluorescence assays detected high PELI1 expression in various cancer cell lines, including Caco-2 epidermoid carcinoma, Hela cervical adenocarcinoma, and U2OS osteosarcoma cells (**Figure 1D**). Additionally, elevated PELI1 levels were observed in clinical specimens from liver, breast, prostate, lung, and colorectal cancers (**Figure 1E**).

### Genetic alterations of PELI1 across tumors

**Figure 2:**
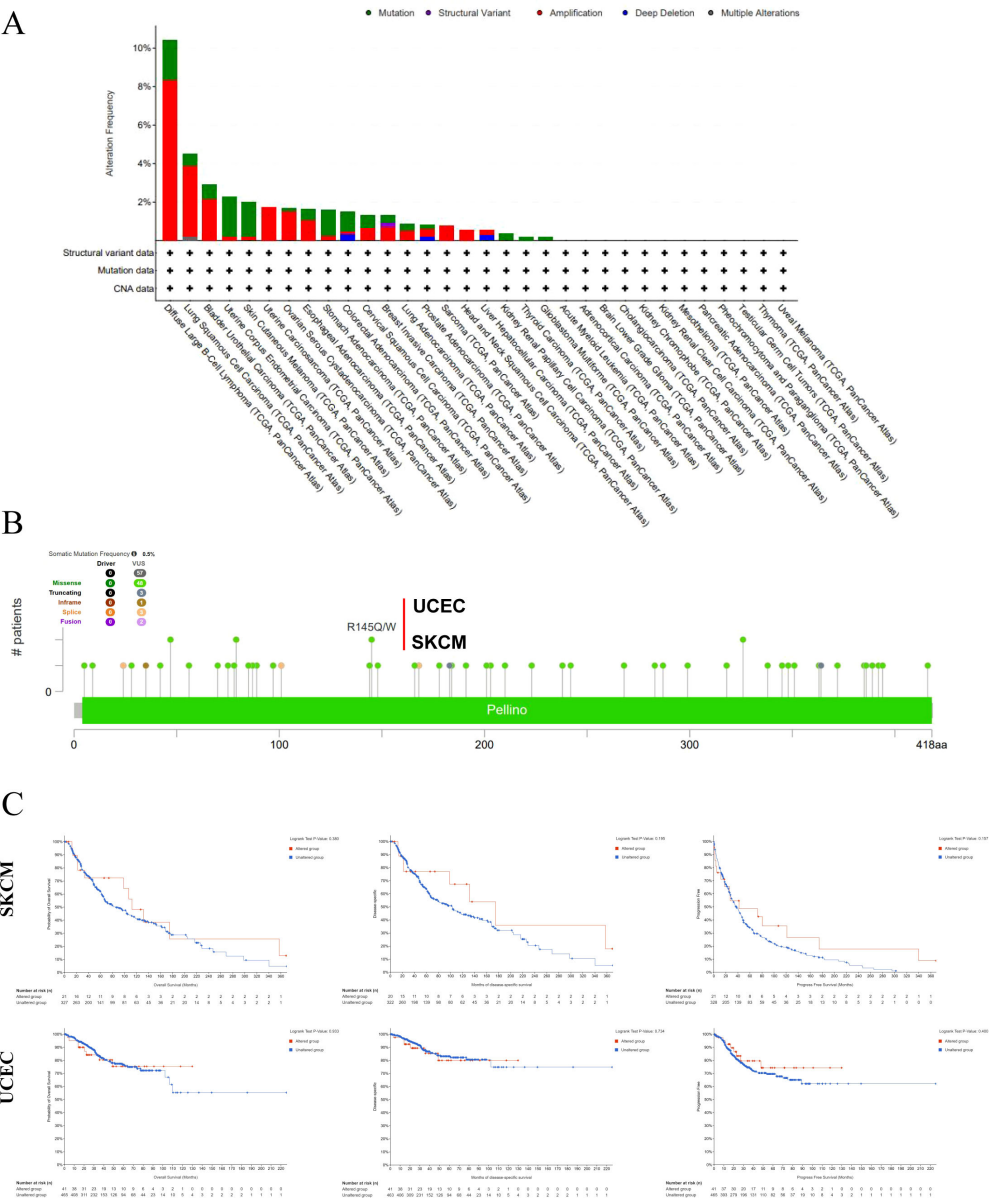
Genetic alteration of PELI1 in different tumors based on The Cancer Genome Atlas (TCGA) database by using the cBioPortal tool. The alteration frequency with mutation type **(A)** and mutation site **(B)** for multiple cancers were shown. The correlation between PELI1 mutation status and overall, disease-specific and progression-free survival were analyzed **(C)**.

**Figure 2A**, obtained from cBioPortal, illustrated the genetic alteration profile of PELI1 in tumor samples. Diffuse large B-cell lymphoma (DLBC) exhibited the highest PELI1 alteration frequency, predominantly involving copy number amplification (CNA). Lung squamous cell carcinoma (LUSC) and bladder urothelial carcinoma (BLCA) also showed substantial PELI1 amplification. **Figure 2B** depicted multiple PELI1 mutation sites in the TCGA cohort, with R145Q/W representing the most frequently altered locus. Two samples from TCGA database harbored this mutation—one from UCEC and another from skin cutaneous melanoma (SKCM). However, the R145Q/W alteration of PELI1 did not significantly affect clinical survival in these UCEC and SKCM cases (**Figure 2C**).

### DNA methylation patterns of PELI1

Compared with normal samples, PELI1 methylation levels showed significant differences in 11 cancer types. Hypermethylation of PELI1 occurred in colon adenocarcinoma (COAD), HNSC, KIRC, LUAD, READ, SKCM, thyroid carcinoma (THCA) and UCEC (**Supplementary Figure S1A**), while hypomethylation was observed in kidney renal papillary cell carcinoma (KIRP), LIHC, prostate adenocarcinoma (PRAD) (**Supplementary Figure S1A**). **Supplementary Figure S1B** displayed heatmaps of the nine cancer types with the most pronounced differences, showing methylation patterns across multiple probes.

### M6A modification analysis associated with PELI1

The N6-methyladenosine (m6A) modification, a prevalent epitranscriptomic regulatory mechanism, is increasingly recognized for its pivotal roles in oncogenesis and malignant tumor progression (19). **Supplementary Figure S2A** presented a heatmap depicting correlations between PELI1 and 21 m6A regulators across diverse cancer types. Notably, PELI1 expression exhibited significant associations with m6A regulators of ovarian cancer (OV), PRAD, pan-kidney cohort (KICH+KIRC+KIRP) KIPAN and HNSC. To further characterize PELI1 regulation by m6A, we bioinformatically predicted m6A modification sites within the PELI1 gene sequence (**Supplementary Figure S2B**). Five high-confidence m6A sites were identified at positions 943, 1338, 1365, 1624 and 1654. Collectively, these findings suggest that m6A regulators may modulate tumor progression across multiple cancer types by regulating PELI1 expression.

### The association between PELI1 expression and prognosis across tumors

**Figure 3:**
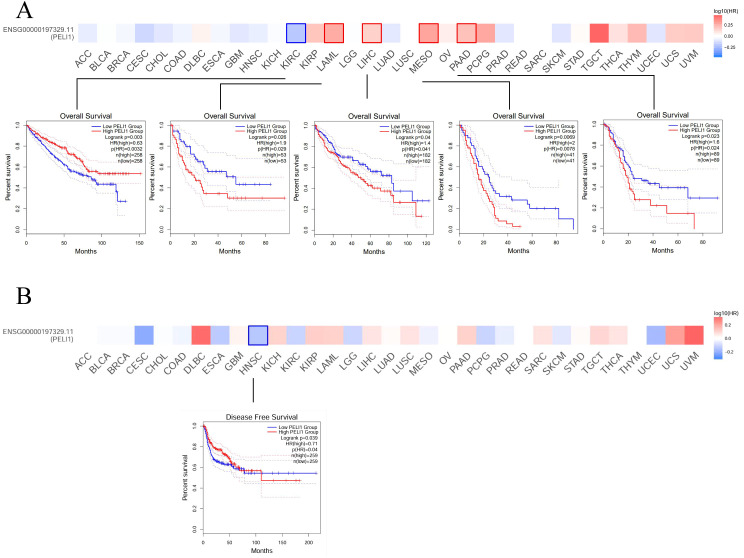
The relationship between PELI1 expression and survival outcome of multiple cancer types. The relationship between PELI1 expression and overall survival outcome of multiple cancer types in The Cancer Genome Atlas (TCGA) database. According to log10 (hazard ratio [HR]), overall survival **(A)** and disease-free survival **(B)** for different patient cohorts were displayed on the survival maps.

To investigate the prognostic value of PELI1 across tumors, Kaplan–Meier survival analysis was performed to evaluate the association between PELI1 expression and clinical outcomes. Our findings revealed that elevated PELI1 expression was consistently associated with worse overall survival (OS) in several tumor types, including leukemia, acute myeloid leukemia (LAML), LIHC, mesothelioma (MESO) and sarcoma (SARC) (**Figure 3A**). Conversely, low PELI1 expression correlated with a unfavorable OS outcomes in KIRC. **Figure 3B** showed elevated PELI1 expression was associated with an unfavorable prognosis, with regards to disease-free survival (DFS), for cancer of HNSC within TCGA database.

### Diagnostic potential of PELI1

The potential diagnostic value of PELI1 in multiple cancers was assessed through receiver operating characteristic (ROC) curve analysis, with the area under the curve (AUC) serving as the primary performance metric. ROC curves were generated for five cancer types—LIHC, KIRC, ESCA, COAD and gastric tumor—based on gene expression data derived from the Gene Expression Omnibus (GEO) datasets, where PELI1 exhibited differential expression levels across these malignancies (**Supplementary Figure S3A-E**). ROC curve analysis based on GEO cohorts for these cancer types revealed that PELI1 achieved robust discriminative power, with AUC values exceeding 0.7 for LIHC (0.7363), KIRC (0.9655), ESCA (0.8048), COAD (0.7500) and gastric tumor (0.8089) (**Supplementary Figure S3A-E**). These results suggest that PELI1 demonstrates promising potential as a novel diagnostic biomarker across diverse malignancies.

### Association of PELI1 expression with tumor mutation burden and microsatellite instability

Elevated tumor mutation burden (TMB) has emerged as a predictive biomarker for response to immune checkpoint inhibitors targeting the programmed cell death 1/programmed death-ligand 1 (PD-1/PD-L1) axis, with its elevation correlating with improved patient survival across diverse malignancies (20). Similarly, microsatellite instability (MSI)—a hallmark of defective DNA mismatch repair—is a well-established biomarker for guiding immune checkpoint blockade (ICB) therapy (21). To explore the relationship between PELI1 expression and these immunotherapy-relevant biomarkers, we analyzed correlations across tumor types. For TMB, PELI1 expression exhibited a positive correlation in ESCA but displayed negative associations in in UCEC, LIHC and LUSC (**Supplementary Figure S4A**). Regarding MSI, PELI1 expression showed positive correlations in testicular germ cell tumors (TGCT) and LAML, whereas negative associations were observed in DLBC, uterine carcinosarcoma (UCS), KIPAN, HNSC, and KICH (**Supplementary Figure S4B**).

### PELI1 expression profiling in LIHC

**Figure 4:**
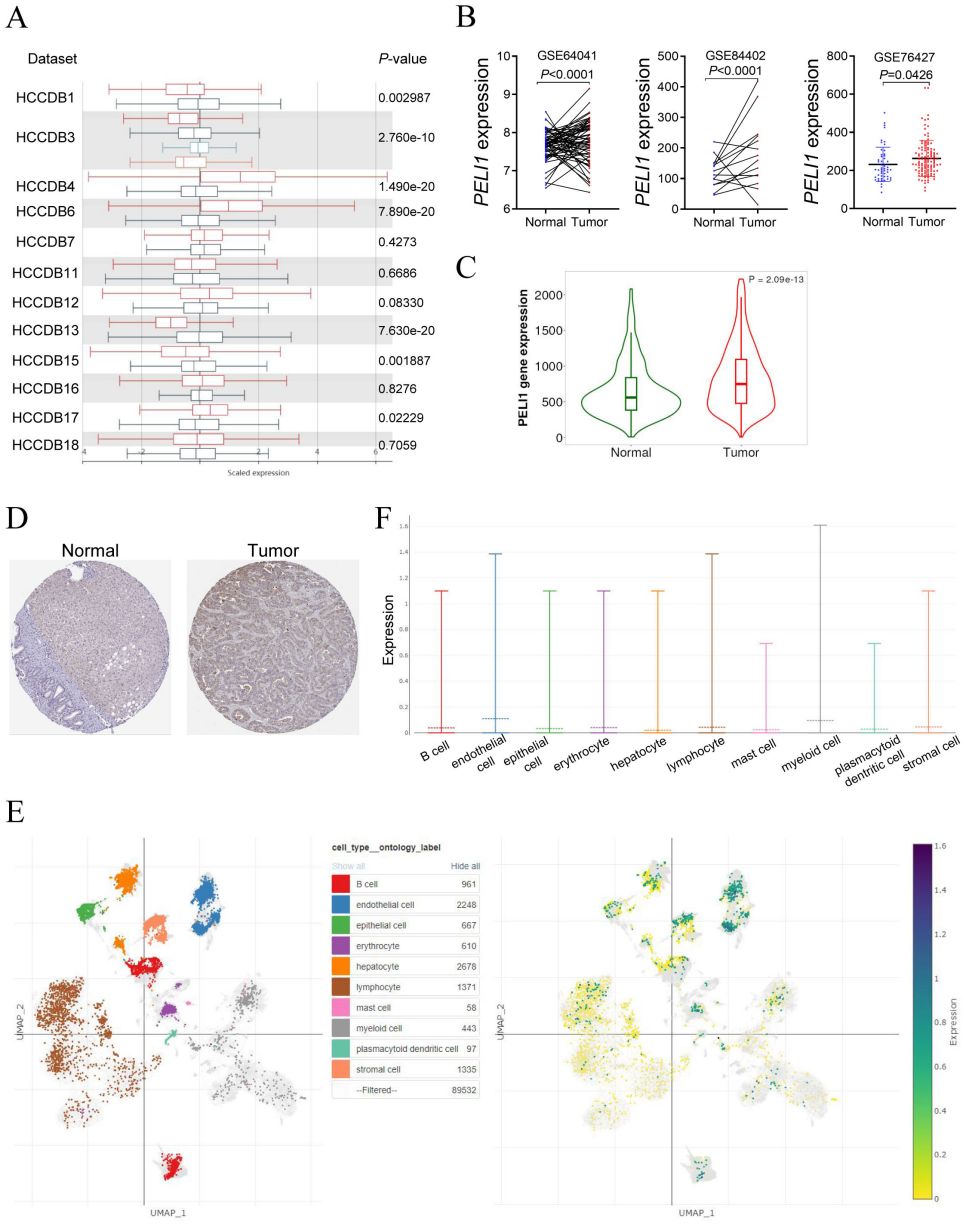
The expression level of PELI1 in human hepatocellular carcinoma. **(A)** PELI1 expression levels in LIHC and normal tissues based on HCCDB database were shown. **(B)** In comparison with normal liver tissues, PELI1 expression levels were significantly upregulated in LIHC tissues in GEO datasets. **(C)** The expression level of PELI1 was higher in LIHC compared with normal tissues from gene chip data of TNMplot database. **(D)** The representative immunohistochemistry images of normal liver tissue and hepatocellular carcinoma tissue in HPA database were shown. **(E)** The single cell sequencing data from hepatocellular carcinoma patients were subjected to dimensionality reduction and clustering. Cell subpopulations were annotated with reference to the CellMarker database (left). The UMAP plot displayed the mRNA expression levels of PELI1 across the various cell subpopulations (right). **(F)** The box plot showed the expression levels of PELI1 across different cell subpopulations in hepatocellular carcinoma patients from panel **(E)**.

Given the robust associations of PELI1 overexpression with key biological and clinical features—including DNA methylation patterns, m6A modification dynamics, patient survival, diagnostic potential, and tumor mutation burden—identified in our prior analyses, we selected LIHC as a representative malignancy for in-depth expression profiling. To validate this association, we analyzed PELI1 expression across 12 HCCDB datasets (HCCDB 1, 3, 4, 6, 7, 11, 12, 13, 15, 16, 17, and 18), which revealed consistent upregulation of PELI1 in HCC tissues relative to adjacent non-tumor tissues (**Figure 4A**). Consistent with these findings, GEO dataset analysis confirmed significant PELI1 upregulation in LIHC tissues versus normal liver controls (**Figure 4B**). Further validation using the TNMplot platform revealed elevated PELI1 mRNA levels in cancerous versus normal tissues across gene chip datasets (**Figure 4C**). Complementing these transcriptomic data, immunohistochemical results from the Human Protein Atlas (HPA) further corroborated this pattern, with protein expression levels of PELI1 aligning with the gene expression trends observed in both tumor and normal tissues (**Figure 4D**). Furthermore, at the single-cell level in hepatocellular carcinoma, PELI1 was highly expressed in endothelial cells, myeloid cells and lymphocyte (**Figures 4E, F**).

### Functional enrichment and co-expression analyses of PELI1

**Figure 5:**
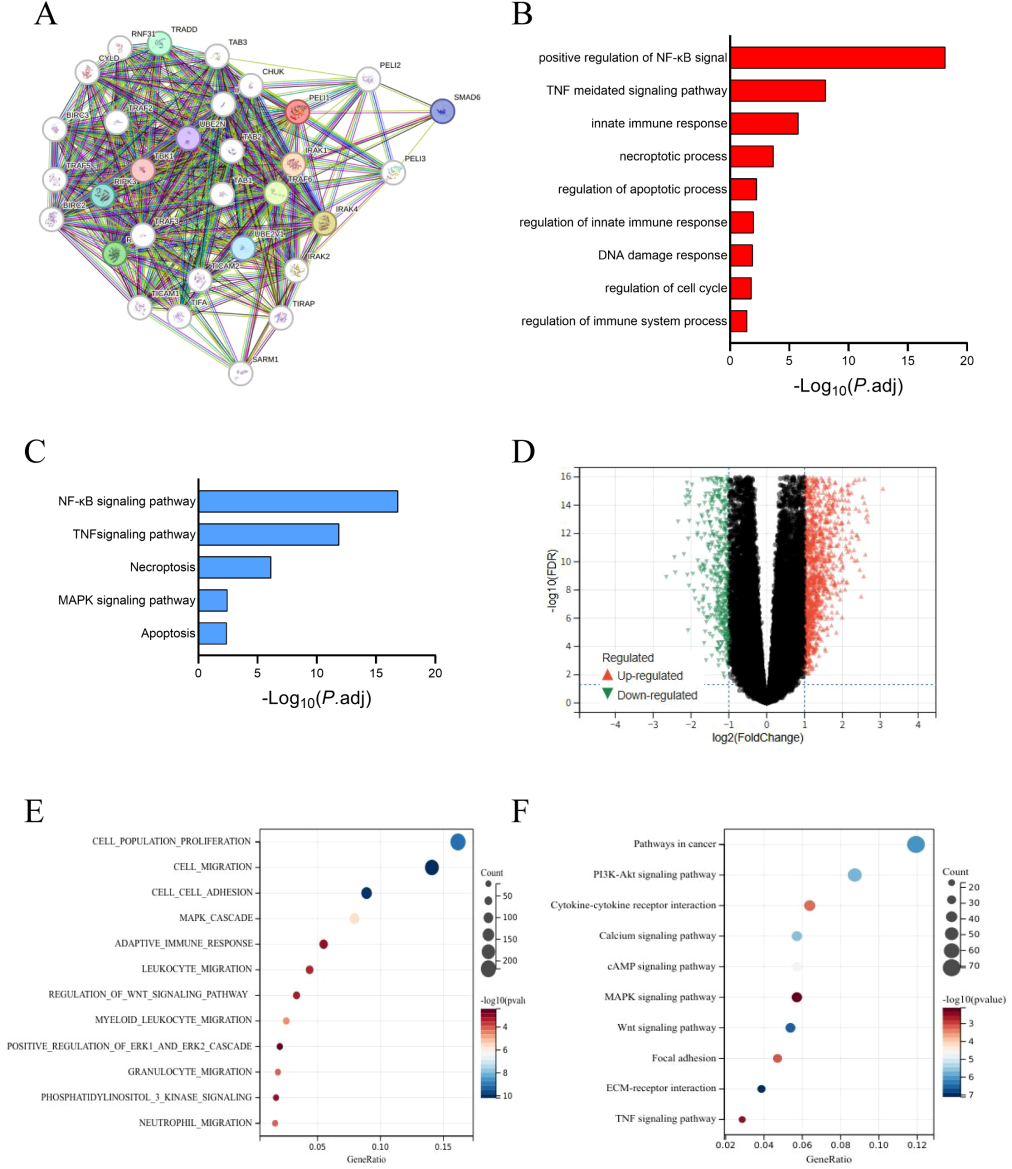
PELI1-related gene set enrichment analysis. **(A)** A series of PELI1-binding proteins supported by experimental evidence were obtained using the STRING tool. **(B, C)** Based on PELI1-binding genes and highly correlated genes, gene ontology (GO) analysis **(B)** and Kyoto Encyclopedia of Genes and Genomes (KEGG) pathway analysis **(C)** were conducted. **(D)** Volcano plot for differential gene expression profiles between PELI1^high^ and PELI1^low^ groups in TCGA cohort. **(E, F)** GO **(E)** and KEGG **(F)** enrichment analysis for differential expressed genes in the volcano plot.

To elucidate the functional roles of PELI1 in LIHC, we performed comprehensive functional enrichment and co-expression analyses. First, we conducted a PELI1-related gene set enrichment analysis and predicted 30 proteins that interact with PELI1 (**Figure 5A**, **Supplementary Table S2**). Next, we integrated LIHC tumor expression data from TCGA to identify the top 100 genes showing significant correlation with PELI1 in LIHC (**Supplementary Table S3**). Functional annotations on both PELI1-binding genes and these co-expressed genes were then performed using Gene Ontology (GO) and Kyoto Encyclopedia of Genes and Genomes (KEGG) pathway analyses. GO analysis revealed that biological processes associated with necroptotic, apoptosis, DNA damage, cell cycle regulation, and immune response were potentially linked to PELI1 function in LIHC (**Figure 5B**). KEGG pathway analysis further highlighted the critical roles of the NF-κB signaling pathway, TNF signaling pathway and MAPK signaling pathway in mediating PELI1’s effects on LIHC progression (**Figure 5C**). To visualize co-expression patterns, we used Linkedomics to generate heatmap representations of the top 50 significantly co-expressed genes (both positively and negatively correlated with PELI1) (**Supplementary Figures S5A, B**). Additionally, Gene Set Enrichment Analysis (GSEA) analysis uncovered additional pathway enrichments regulated by PELI1 and its co-expressed genes, including mitochondrial respiratory chain complex assembly, cellular amino acid metabolic process, TNF signaling pathway and focal adhesion (**Supplementary Figures S5C**, **D**). Regulatory network analysis identified the most significant downstream regulators: MAPK3 as the key kinase target, MIR-410 as the prominent miRNA target, and V$EVI1_06 as the dominant transcription factor (TF) target (**Supplementary Table S4**). To further characterize the transcriptional landscape associated with PELI1 expression, we performed a differential gene expression analysis between the PELI1^high^ and PELI1^low^ groups using a volcano plot (**Figure 5D**). This analysis revealed 1,604 upregulated genes and 584 downregulated genes in the PELI1^high^ group (**Supplementary Table S5**). Functional annotation of these differentially expressed genes showed enrichment in immune-related biological processes, including leukocyte migration, myeloid leukocyte migration, granulocyte migration and neutrophil migration involved in the immune response among GO terms (**Figure 5E**). At the pathway level, KEGG enrichment highlighted the PI3K-AKT signaling pathway, MAPK signaling pathway, Wnt signaling pathway, and TNF signaling pathway (**Figure 5F**).

### Correlation between PELI1 expression and the tumor immune landscape

**Figure 6:**
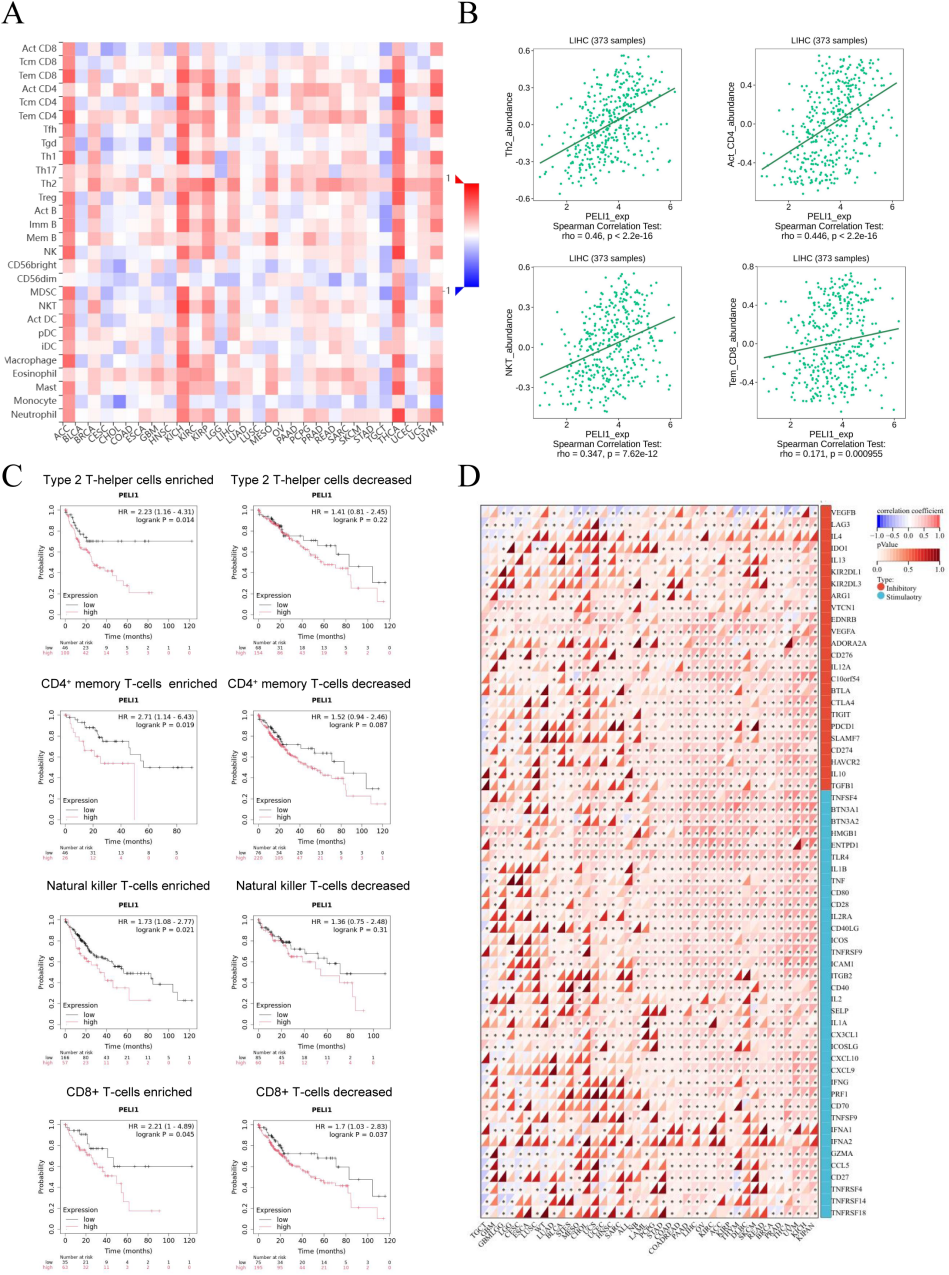
The correlation between immune cell infiltration and PELI1 expression. **(A)** Heatmap of the correlation between PELI1 expression and 28 subtypes of tumor infiltrating immune cells across multiple human cancer types including LIHC. **(B)** PELI1 expression was associated with infiltrating immune cell subtypes in LIHC. **(C)** Correlations between PELI1 expression and OS in different immune cell subgroups in LIHC patients were determined by Kaplan–Meier survival plotter. **(D)** Correlation analysis of PELI1 expression and immune checkpoint molecules across multiple human cancer types including LIHC.

Tumor-infiltrating lymphocytes are crucial drivers of tumorigenesis and significantly affect patient prognosis (22). Given the functional enrichment results highlighting PELI1’s involvement in immune-related processes (e.g., immune response, leukocyte migration), we hypothesized that PELI1 expression might correlate with immune cell infiltration levels in tumors. To test this, we first evaluated the association between PELI1 expression and the abundance of infiltrating immune cell subsets across multiple tumor types. Our analysis revealed a positive correlation between PELI1 expression and the infiltration of multiple immune cell populations, including CD4(+) T (CD4 T) cells, Th2 cells, eosinophils, mast cells, and neutrophils (**Figure 6A**). Notably, in LIHC specifically, PELI1 expression exhibited a strongly positive correlation with the enrichment of Th2 cells, activated CD4 T cells, NKT cells and effector memory CD8 T cells (**Figure 6B**, **Supplementary Figure S6**). Conversely, PELI1 expression was negatively correlated with the infiltration of CD56dim NKT cells (**Supplementary Figure S6**). These findings collectively support an association between PELI1 and the immune microenvironment in LIHC. To further explore the clinical implications of this relationship, we investigated how PELI1 expression and immune cell infiltration jointly impact tumor survival. Using Kaplan–Meier survival analysis stratified by PELI1 expression levels in LIHC patients with high infiltration of specific immune cell subsets (Th2 cells, CD4 memory T cells, NKT cells and CD8 T cells), we observed that higher PELI1 in LIHC tumors in enriched CD8 T cells correlated with worse patient prognosis (**Figure 6C**). Given PELI1’s potential role as an oncogene in LIHC, we next examined its association with immune checkpoints molecules, which are key regulators of T cell activation and tumor immune evasion. Strikingly, PELI1 expression exhibited significant positive correlations with the expression levels of immune checkpoint genes, including PDCD1, CD274, HAVCR2, LAG3, TIGIT, and CTLA4, in LIHC (**Figure 6D**).

**Figure 7:**
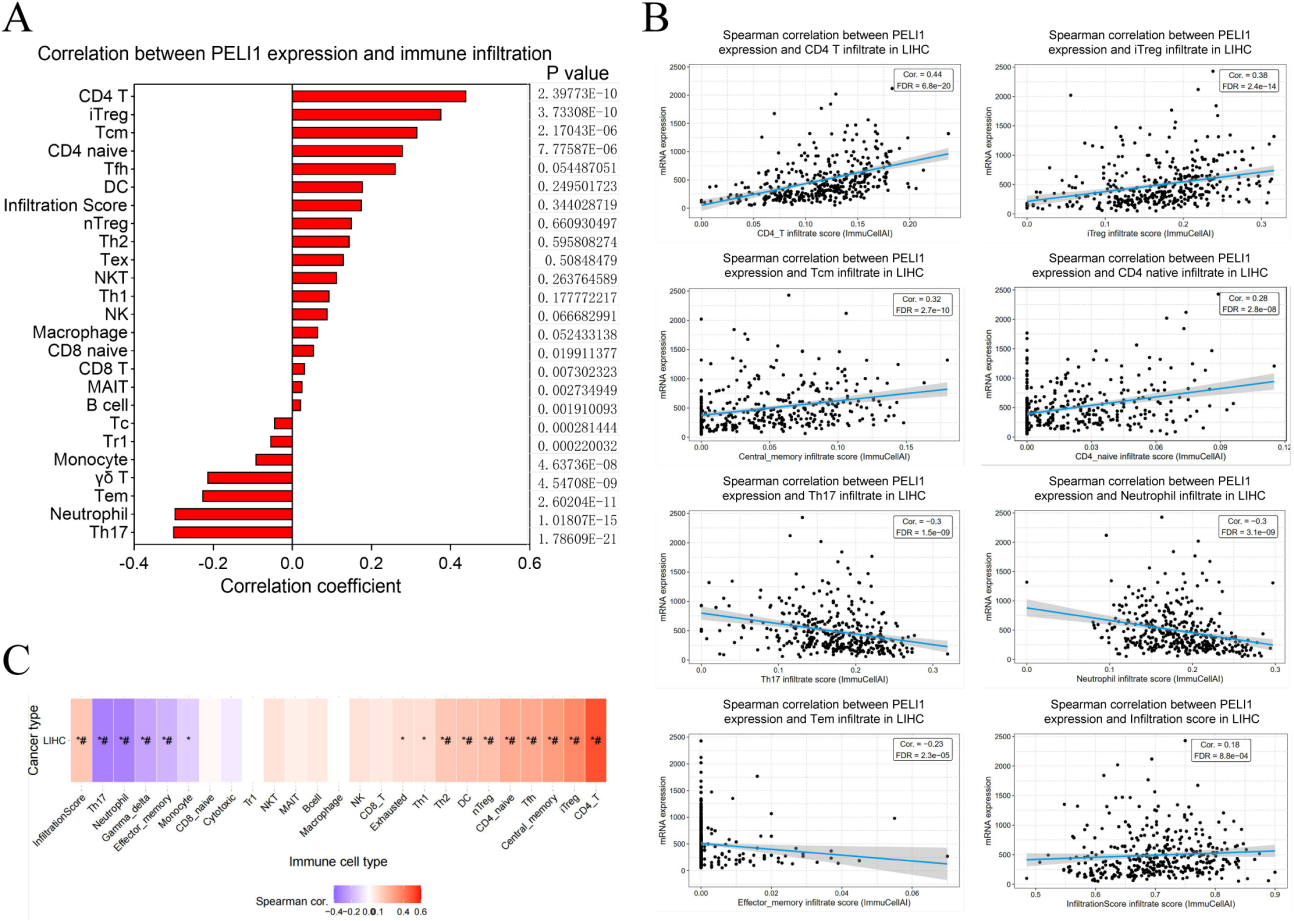
Association between PELI1 expression and immune cell abundance according to GSCA. **(A)** The correlation between PELI1 and immune cell infiltration using ssGSEA in LIHC. **(B)** Spearman correlations of PELI1 expression with significant immune infiltration types in LIHC. **(C)** The association between immune cell infiltrates and GSVA score in LIHC. *P < 0.05; ^#^FDR < 0.05.

To complement these findings, we used the single-sample gene set enrichment analysis (ssGSEA) algorithm (via the GSCA database) to assess immune infiltration levels across diverse immune cell types. PELI1 expression was positively correlated with the infiltration levels of CD4 T cells, induced regulatory T cells (iTreg cells), central memory T (Tcm) cells, and CD4(+) naive (CD4 naive) cells, while it was negatively correlated with the infiltration levels of Th17 cells, neutrophils, effective memory T (Tem) cells, γδ T cells and monocytes in LIHC (**Figure 7A**). Spearman correlation coefficients for the most strongly associated immune cell infiltrates—including CD4 T cells, iTreg cells, Tcm cells, CD4 naive cells, Th17 cells, neutrophils, Tem cells, and total infiltration score—were also displayed (**Figure 7B**). Finally, a heatmap summarized the correlations between immune cell infiltration levels and GSVA enrichment scores, which represents the gene set expression level of LIHC (**Figure 7C**). To evaluate the therapeutic relevance of PELI1, we analyzed its association with drug response in the CellMiner database. A significant positive correlation was identified between PELI1 expression and the IC50 of CFI-402257 (r = 0.0992, P = 0.0373; **Supplementary Figure S7**), suggesting that LIHC patients with high PELI1 expression may exhibit reduced sensitivity to this compound.

### The biological functions of PELI1 in LIHC

**Figure 8:**
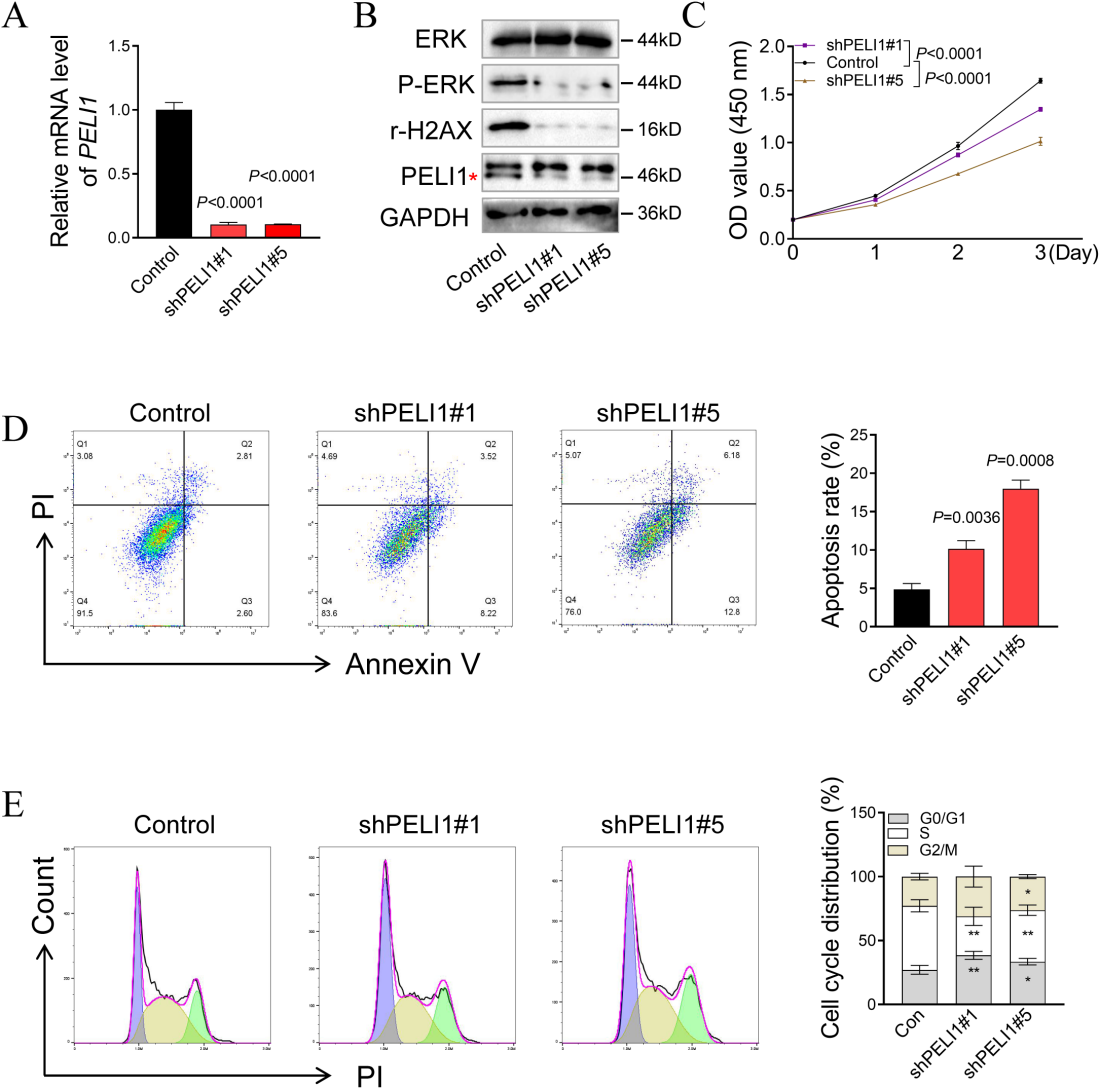
The biological functions of PELI1 in human hepatocellular carcinoma. **(A)** Quantification of mRNA expression of *PELI1* in HepG2 cells transduced with retroviruses encoding shPELI1 or shNC. shNC represents a non-targeting shRNA. **(B)** Immunoblotting analysis of indicated protein levels in HepG2 cells transduced with retroviruses encoding shPELI1 or shNC. GAPDH was used as a loading control. **(C)** Statistical analysis of cell proliferation in the HepG2 cells transduced with retroviruses encoding indicated shRNA. **(D, E)** The cell apoptosis **(D)** and cell cycle **(E)** were measured in the HepG2 cells transduced with retroviruses encoding indicated shRNA by flow cytometry. Data are presented as mean ± SD from three independent experiments. *P<0.05; **P<0.01.

For biological validation, we selected HepG2 cells—an established *in vitro* model for LIHC—owing to their extensively characterized genetic and phenotypic relevance to human disease. To assess the functional impact of PELI1, we transfected HepG2 cells with PELI1-targeting short hairpin RNAs (shRNAs) to achieve specific knockdown, which was confirmed by RT-qPCR and western blotting (**Figures 8A, B**). Functional assays revealed that PELI1 knockdown significantly suppressed the proliferation of HepG2 cells (**Figure 8C**). Mechanistic investigations via flow cytometry analysis further demonstrated that PELI1 knockdown promoted apoptosis (**Figure 8D**) and triggered G1-phase cell cycle arrest in HepG2 cells (**Figure 8E**). Notably, western blotting analysis highlighted concomitant downregulation of p-ERK and r-H2AX in PELI1-knockdown HepG2 cells (**Figure 8B**), suggesting potential molecular links between PELI1 signaling and these phenotypic changes.

## Discussion

Tumors pose a substantial and life-threatening challenge to global health. Despite significant advancements in diagnosis and treatment, the 5-year overall survival rate for most malignancies remains disappointingly low (23), underscoring the urgent need for innovative diagnostic strategies and therapeutic targets. The TCGA database has revolutionized cancer research by integrating multi-omics data to systematically analyze 33 prevalent tumor types, providing an unparalleled resource for exploring gene functions across diverse cancers (24, 25). With the rapid development of bioinformatics tools and public databases, recent years have witnessed growing efforts to identify and characterize pan-cancer molecular biomarkers and their functional roles (26, 27). In this study, we systematically performed a comprehensive analysis of PELI1 across multiple tumor types using publicly available datasets to elucidate its expression patterns, clinical relevance, and oncogenic mechanisms, with further in-depth validation in LIHC.

By analyzing GEO and TCGA datasets, we observed significant upregulation of PELI1 in multiple malignancies, including CHOL, ESCA, KIRC, LIHC and STAD. This finding is consistent with recent experimental studies demonstrating that PELI1 overexpression promotes tumor progression in breast cancer, lung cancer, and lymphoma (6). To evaluate the prognostic significance of PELI1, we correlated its expression with clinical outcomes across different cancers. Kaplan–Meier survival analysis demonstrated that high PELI1 expression was associated with unfavorable survival outcomes in patients with LAML, LIHC, MESO, SARC, KIRC and HNSC. Moreover, ROC curve analysis identified PELI1 as a highly accurate diagnostic marker for several tumor types, including LIHC, KIRC, ESCA, COAD and gastric tumor. Previous studies have reported that PELI1 can serve as a prognostic biomarker in cancers such as diffuse large B-cell lymphoma and pancreatic cancer (28, 29). Together, these findings not only confirm PELI1 overexpression in tumors but also highlight its potential as both a prognostic and diagnostic biomarker. Although our bioinformatic analyses strongly suggest the biomarker potential of PELI1, its clinical applicability requires further validation in prospective, large-scale cohorts. Future efforts should focus on establishing standardized immunohistochemical (IHC) assays for PELI1 detection using tumor tissue microarrays and evaluating its diagnostic and prognostic utility in a real-world clinical setting, ideally in combination with established biomarkers to assess its additive value.

Building on these observations, we focused on LIHC to elucidate the downstream oncogenic mechanisms of PELI1. Functional enrichment analysis of PELI1-bound and coexpressed genes revealed that coexpressed genes with PELI1 were primarily enriched in pathways related to apoptosis, cell cycle progression, and DNA damage response. These results suggest PELI1 may promote tumor growth by regulating apoptotic and cell cycle processes. To validate its functional role, we performed systematic phenotypic characterization of PELI1-knockdown HepG2 cells. PELI1 suppression significantly promoted apoptosis, impeded G1-phase cell cycle progression, and ultimately inhibited cell proliferation. Concurrently, PELI1 knockdown markedly suppressed the protein expression of r-H2AX, potentially attributed to cell cycle arrest and reduced replication stress. KEGG enrichment analysis further highlighted PELI1-mediated pathways, including PI3K-AKT, MAPK, Wnt and TNF signaling—pathways well-characterized in LIHC for targeted anti-cancer drug development (30). Biological validation further confirmed MAPK-ERK pathway regulation by PELI1 in LIHC. Furthermore, regulatory network analysis identified MAPK3 as a key kinase target of PELI1. MAPK3, a member of the MAPK family, encodes extracellular signal-regulated kinase 1 (ERK1), which is involved in various biological processes including cell proliferation, differentiation, apoptosis, and cell cycle regulation (31). We hypothesize that PELI1 suppression significantly promotes apoptosis, impedes cell cycle progression, and ultimately inhibits cell proliferation by targeting the key kinase MAPK3, thereby inhibiting the MAPK-ERK pathway. Although previous studies have established PELI1 as an oncogene in malignancies such as breast cancer, lung cancer, and lymphoma—primarily through its modulation of the PI3K/AKT and B-cell signaling pathways (32, 33)—our findings uncovered a previously uncharacterized role and mechanism for PELI1 in LIHC. Such functional divergence may reflect tissue-specific signaling contexts, highlighting the complexity of PELI1 in cancer biology. Thus, our work not only reinforces the oncogenic role of PELI1 but also broadens its functional repertoire by delineating a hepatocellular carcinoma -specific mechanistic pathway.

However, the specific mechanism by which PELI1 regulates MAPK3 remains unclear. Studies have shown that the E3 ubiquitin ligase PELI1 primarily promotes target protein stability by mediating K63-linked ubiquitination, or alternatively, degrades target proteins through K48-linked ubiquitination (6). In our study, we found that inhibition of PELI1 significantly downregulated ERK1 protein expression, suggesting that PELI1 may stabilize ERK1 by mediating its K63-linked ubiquitination, thereby regulating the MAPK-ERK signaling pathway in LIHC. This hypothesis, however, requires further experimental validation.

Mounting evidence underscores the critical role of the tumor immune microenvironment (TIME) in tumorigenesis and progression (34). While PELI1 has been linked to immune infiltration in the TIME, prior studies have only focused on lymphoma (33) and atherosclerosis (35). Our analysis revealed a positive correlation between PELI1 expression and most infiltrated immune cells, including Th2 cells, activated CD4 T cells, NKT cells and effector memory CD8 T cells, suggesting PELI1 may drive immune infiltration in LIHC. A strong positive correlation between infiltration scores and PELI1 expression further supports its role in shaping the TIME. Additionally, single-cell sequencing analysis identified specific overexpression of PELI1 in myeloid and lymphoid cells in LIHC. Previous studies have shown that PELI1 negatively regulates T cell activation, thereby facilitating T cell anergy and self-tolerance (36). Based on these, we propose a dual mechanism through which PELI1 fosters an immunosuppressive TIME: by facilitating the development of immunosuppressive M2-type tumor-associated macrophages (TAMs) in myeloid cells and directly instigating T cell anergy in lymphocytes. These actions collectively paralyze the anti-tumor immune response, culminating in immune evasion and hepatocellular carcinoma progression. To explore potential clinical implications, we analyzed correlations between PELI1 and immune checkpoint molecules in LIHC. We showed that PELI1 expression was positively associated with checkpoints including PDCD1, CD274, HAVCR2, LAG3, TIGIT, CTLA4. Notably, several of these checkpoints (e.g., PDCD1, LAG3, TIGIT and CTLA4) collectively impair T cell function through distinct mechanisms, driving immune evasion and tumor progression (37). However, the molecular interplay between PELI1 and these checkpoints remains unclear and warrants further investigation. Collectively, our findings suggest PELI1 may modulate the TIME and hold potential as a target for novel immunotherapeutic strategies.

The findings of this study not only reveal a novel mechanism of PELI1 in LIHC but also suggest potential pathways for its clinical translation. First, the significant correlation between PELI1 expression and patient prognosis indicates that it could serve as a valuable prognostic biomarker. In the future, an immunohistochemical assay targeting PELI1 could be developed for clinical risk stratification. Second, while our cellular and clinical data strongly suggest PELI1’s therapeutic potential, future studies using PELI1-specific inhibitors in animal models are warranted to fully confirm its translational value. The development of highly selective small-molecule inhibitors or PROTAC degraders targeting PELI1 may offer an innovative therapeutic strategy for LIHC. Most importantly, our data suggests an association between PELI1 expression and sensitivity to CFI-402257, leading us to propose a potential combination therapy approach: co-administration of a PELI1 inhibitor and CFI-402257 could simultaneously target multiple synergistic pathways, potentially overcoming the limitations of monotherapy and enhancing antitumor efficacy in LIHC. Certainly, these hypotheses will require further validation through extensive preclinical studies and subsequent clinical trials.

## Conclusion

Our systematic pan-cancer analysis reveals the diagnostic, prognostic, and oncogenic roles of PELI1, with in depth mechanism verification in LIHC, nominating it as a promising therapeutic target in LIHC. These findings pave the way for future mechanistic studies and guide the development of PELI1-targeted therapies. Nonetheless, this study has certain limitations. First, our data are subject to inherent heterogeneity from public databases, due to variations in sample processing sequencing platforms, sample size and clinical characteristics. Second, future investigations employing *in vivo* models, such as xenograft experiments in immunodeficient mice, are essential to validate the impact of PELI1 on LIHC tumor growth and metastasis in a complex organismal context. Additionally, the interplay between PELI1 and immune checkpoints in LIHC requires further study to clarify its mechanism and clinical significance. Future work must integrate multi-omics data with functional assays to define PELI1’s context-specific mechanisms. Pursuing PELI1 as a biomarker for patient stratification or combination therapy would accelerate its clinical translation. By addressing these gaps, our work will pave the way for precise PELI1-targeted cancer therapies.

## Data Availability

The original contributions presented in the study are included in the article/**supplementary material**. Further inquiries can be directed to the corresponding authors.
