# Supplementary material for: PELI1 in human cancers: a pan-cancer exploration of its molecular function, clinical significance, and immunomodulatory roles
Source: Front Immunol. 2026 Jan 5;16:1682086. doi: 10.3389/fimmu.2025.1682086 (PMC12813145; doi:10.3389/fimmu.2025.1682086)
Supplement: Supplementary file 1 [file Table1.docx]

Supplementary Material

**Supplementary Figures and Tables**

**
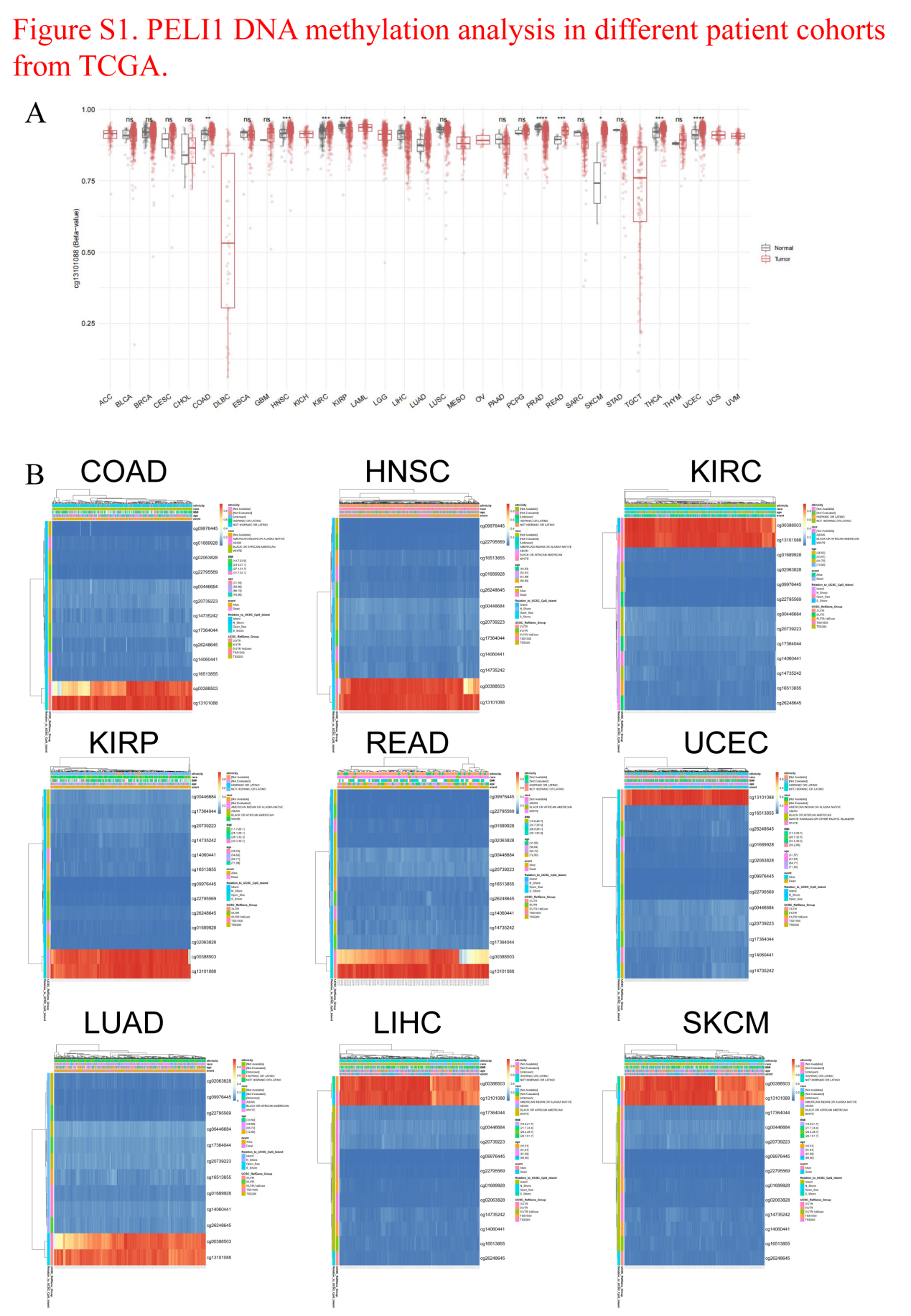
**

**Figure S1. DNA methylation analysis of PELI1 in different patient cohorts from TCGA.** (**A**) The methylation level of PELI1 was analyzed in different tumors from TCGA database. (**B**) The 8 cancer types with the most significant differences in (**A**) were selected to draw heatmaps for multiple probes. ns: not significant. * *P* < 0.05; ** *P* < 0.01; *** *P* < 0.001; **** *P* < 0.0001.


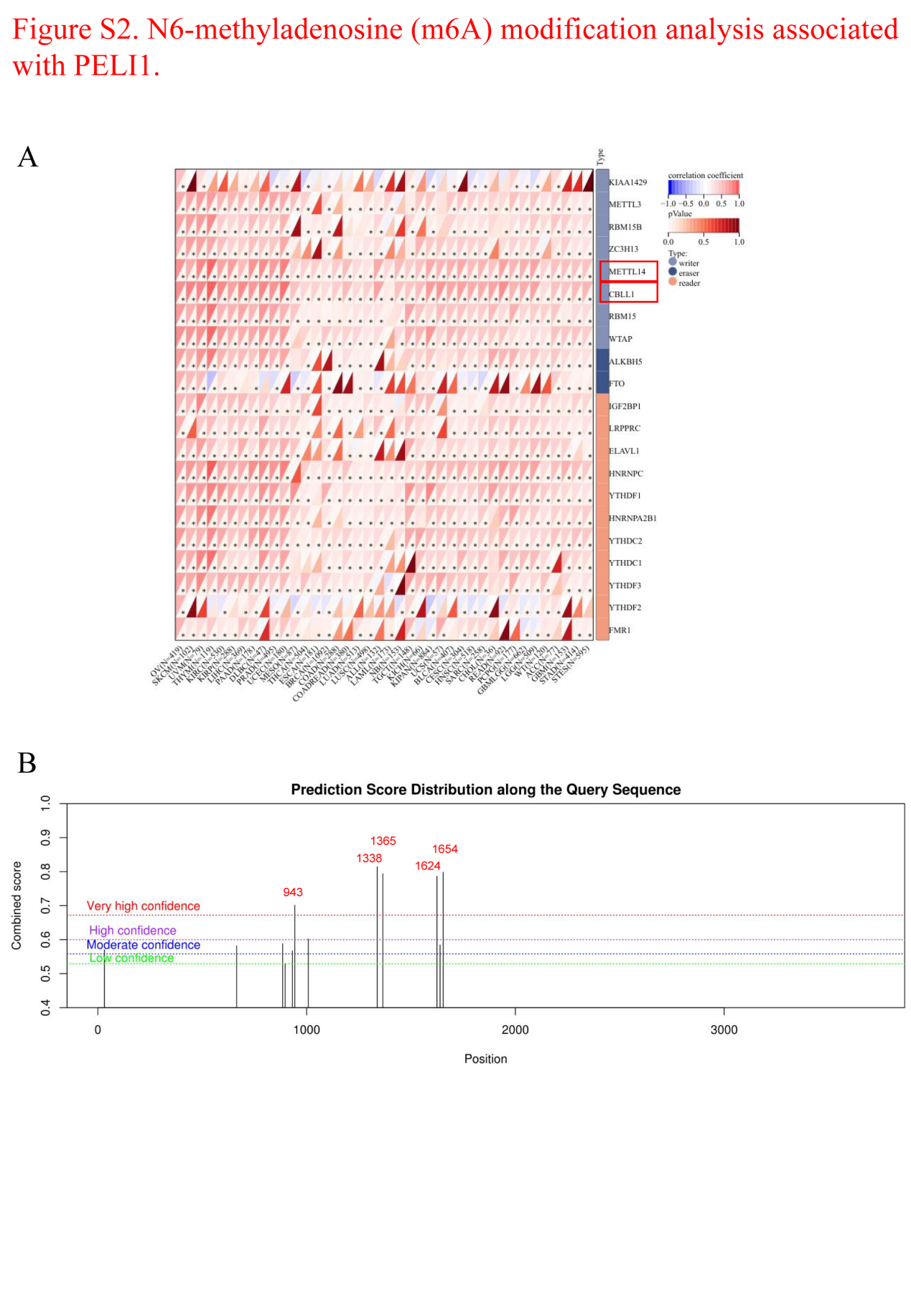


**Figure S2. N6-methyladenosine (m6A) modification analysis associated with PELI1.** (**A**) The correlation analysis between PELI1 and 21 m6A regulators in different cancer types based on The Cancer Genome Atlas (TCGA) database were shown in the corresponding heatmap. (**B**) The computational identification of m6A modification sites were shown for PELI1 sequence.


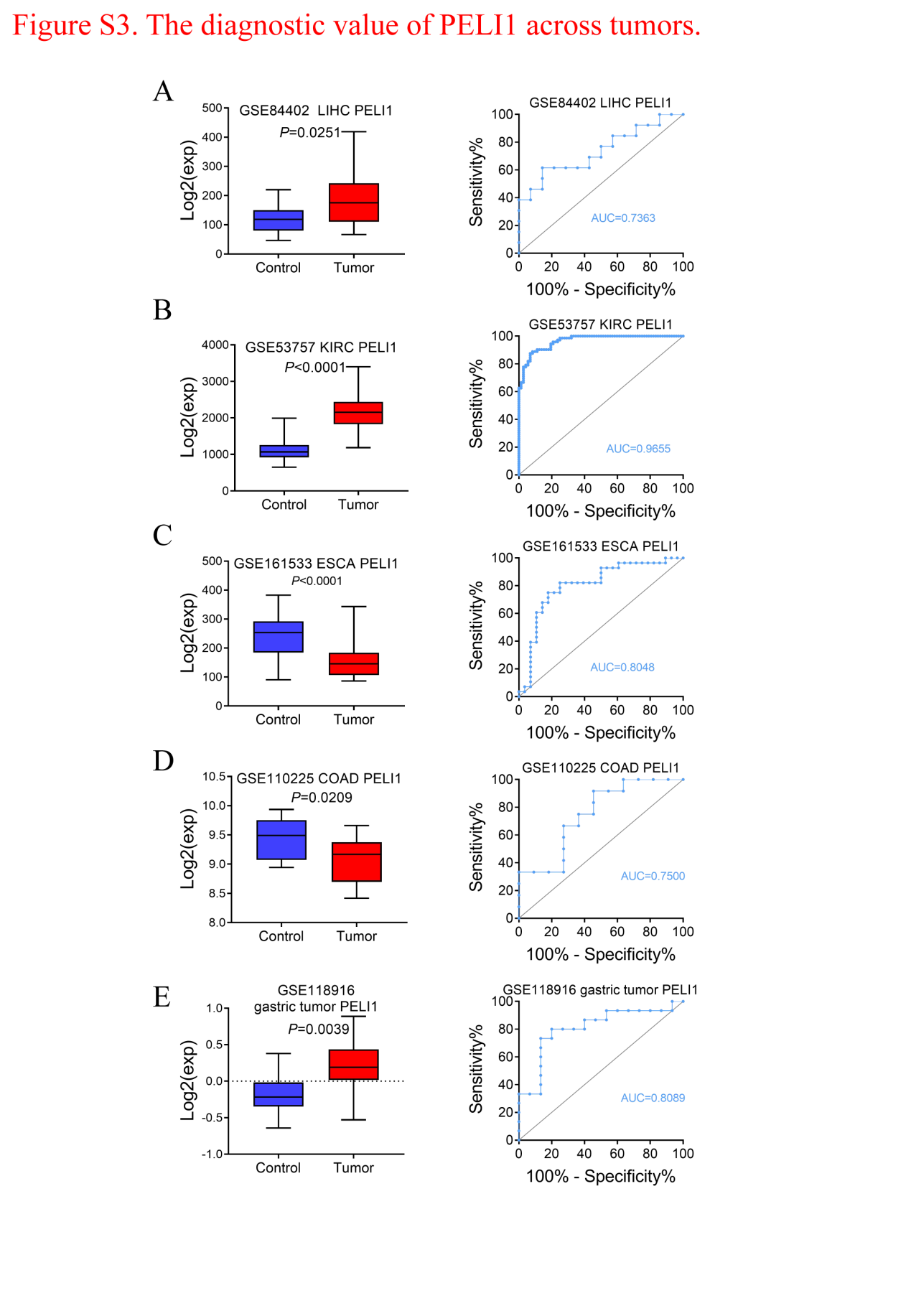


**Figure S3. The diagnostic value of PELI1 across tumors.** Through receiver operating characteristic (ROC) curve analysis, the potential diagnostic value of PELI1 was assessed for five cancer types, including LIHC (**A**), KIRC (**B**), ESCA (**C**), COAD (**D**) and gastric tumor (**E**). Each set of figures include expression profile of PELI1 and ROC curve based on GEO cohort.

**
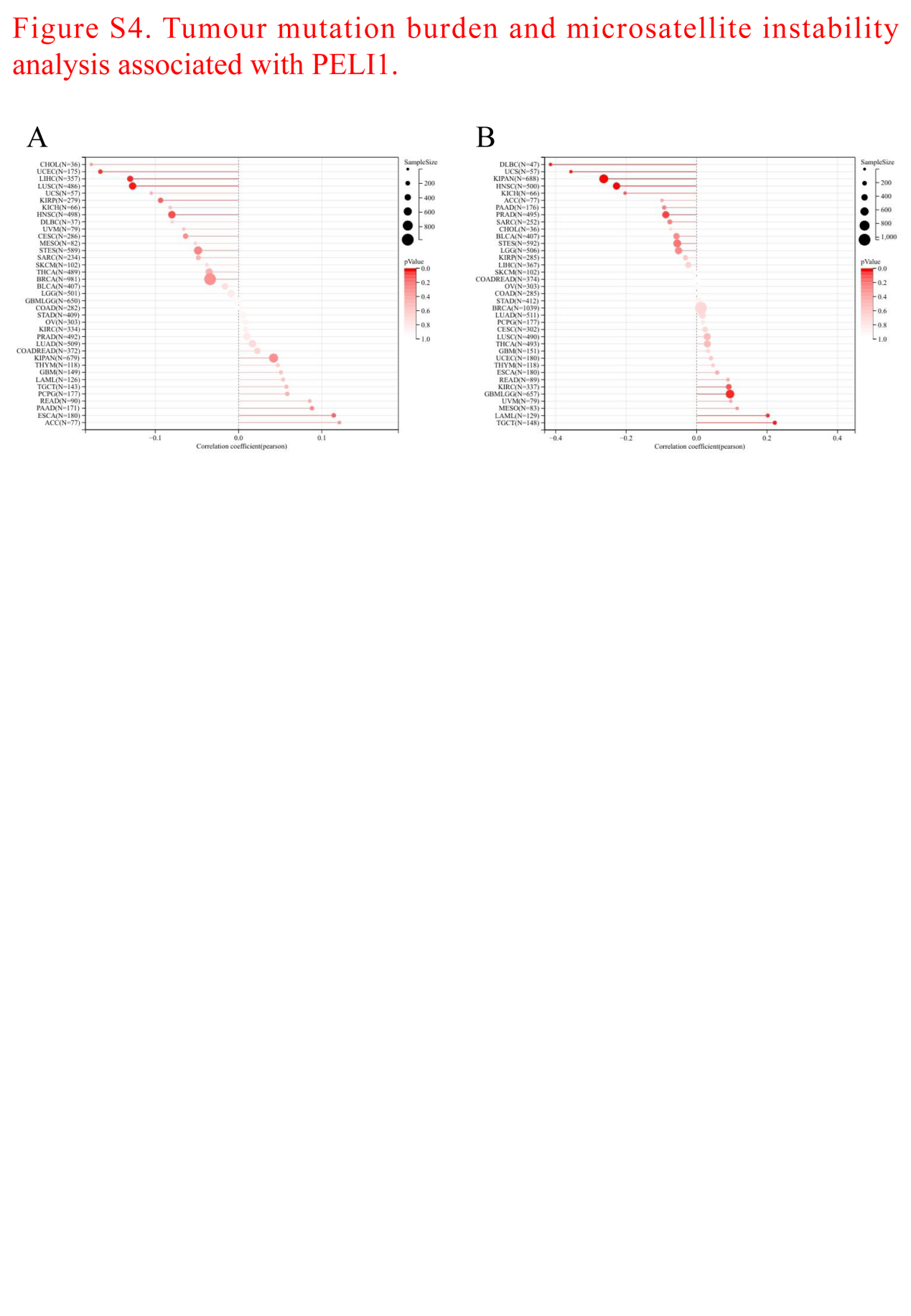
**

**Figure S4. Tumor mutation burden and microsatellite instability analysis associated with PELI1.** (**A-B**) The correlation between PELI1 expression and tumor mutation burden (TMB) (**A**) and microsatellite instability (MSI) (**B**) across all tumors of TCGA.


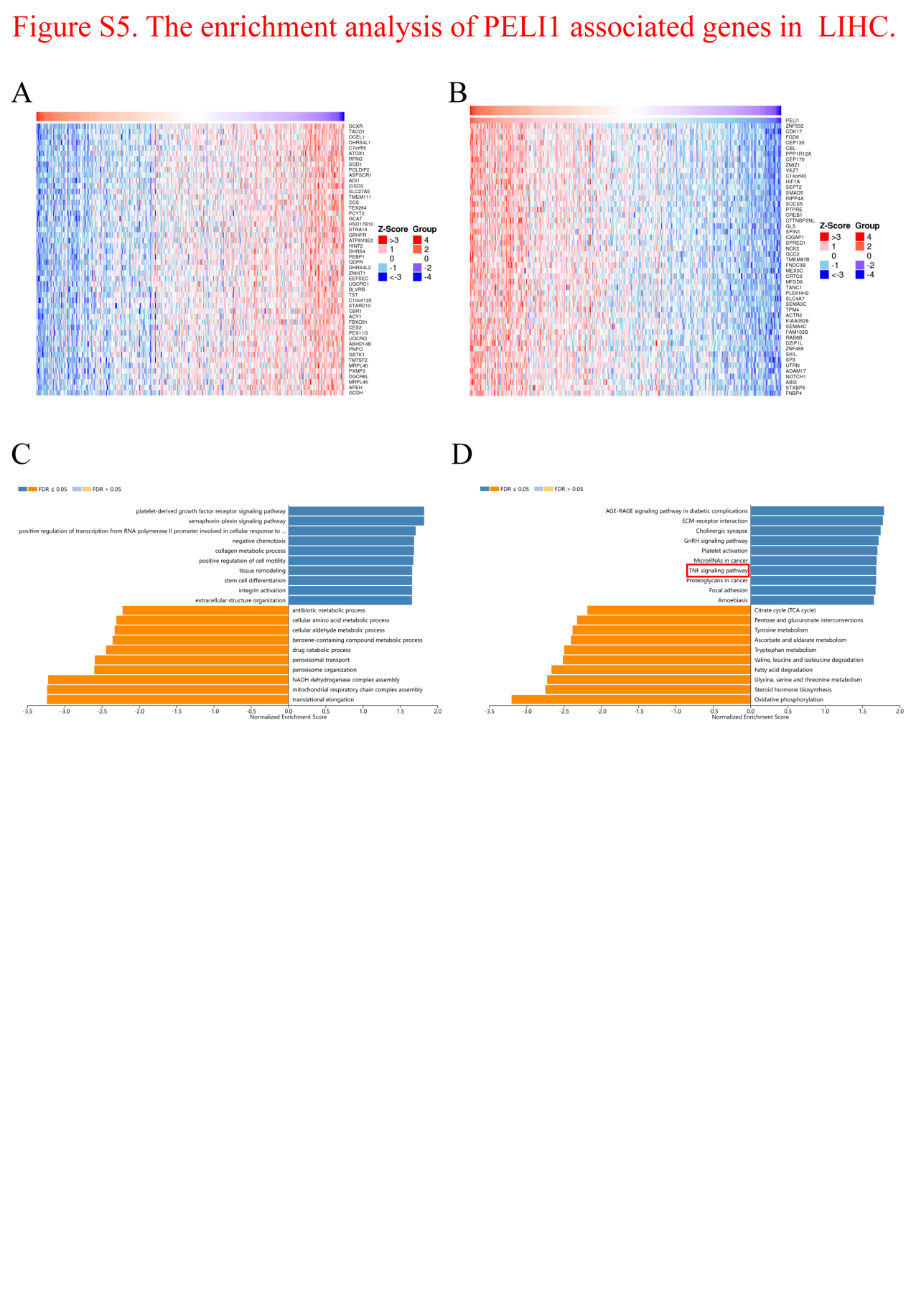


**Figure S5. The enrichment analysis of PELI1 associated genes in LIHC.** (**A-B**) The top 50 genes positively (**A**) and negatively (**B**) correlated to PELI1. (**C-D**) GO (**C**) and KEGG (**D**) analysis of PELI1 co-expression genes.


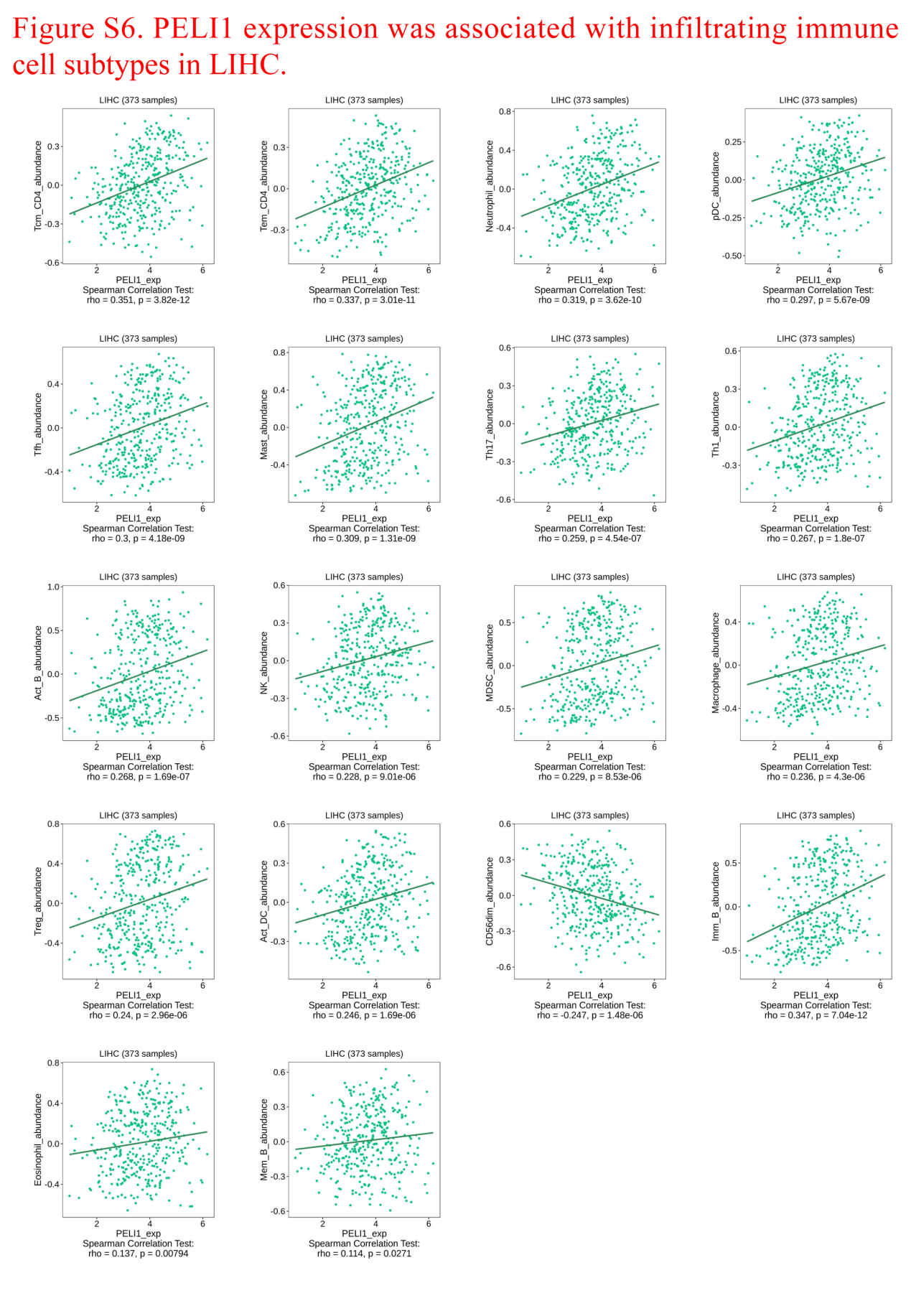


**Figure S6. The association between PELI1 expression and infiltrating immune cell subtypes in LIHC.**

**
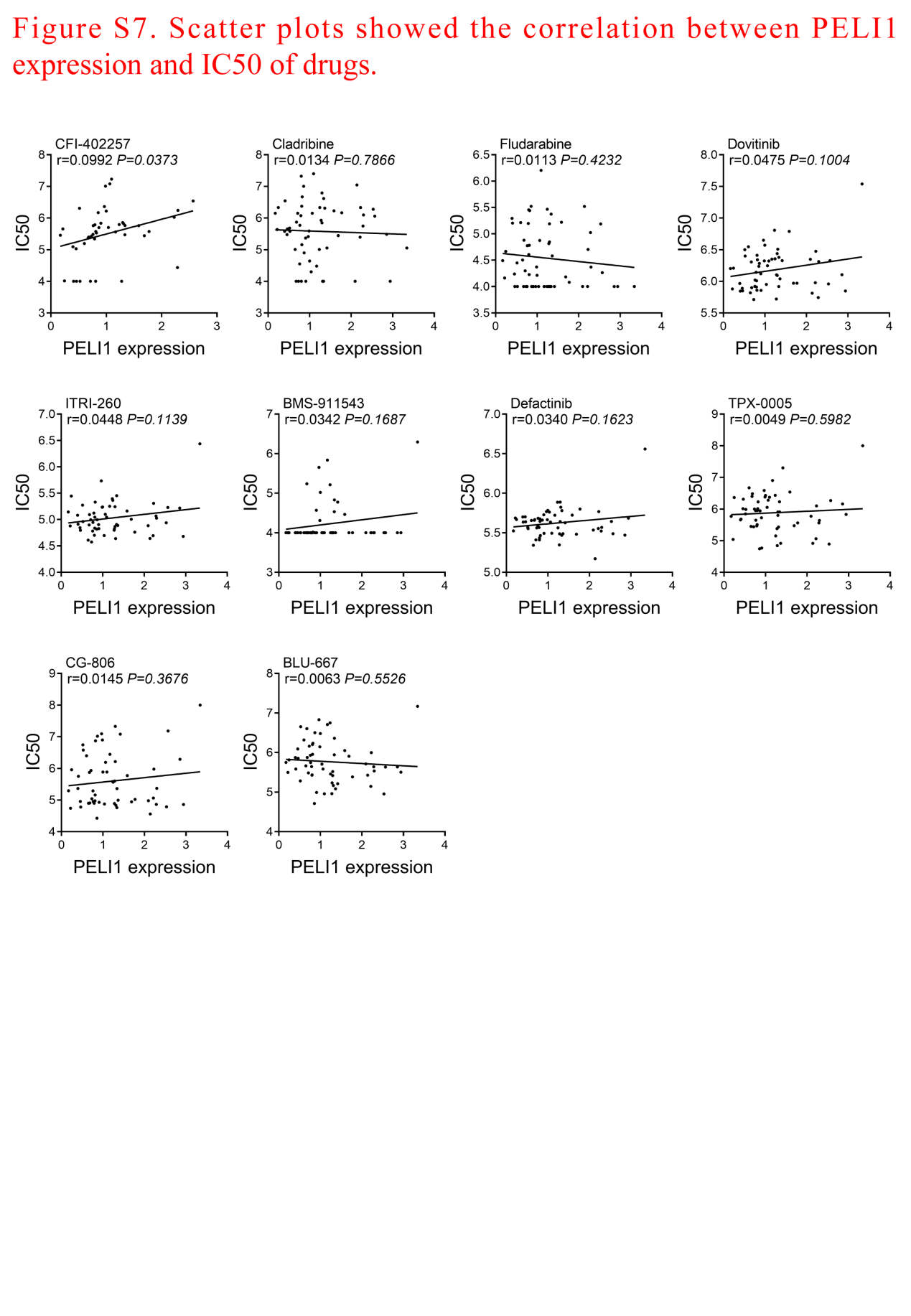
**

**Figure S7. Scatter plots showed the correlation between PELI1 expression and IC50 of drugs.**

**Table S1. PELI1 expression in tumor samples from TCGA cancer types.**

| **Tumor type** | **Abbreviation** | **Expression level compared with normal samples** |
| --- | --- | --- |
| Bladder Urothelial Carcinoma | BLCA | ns |
| Breast invasive carcinoma | BRCA | downregulated |
| Cervical squamous cell carcinoma and endocervical adenocarcinoma | CESC | ns |
| Cholangiocarcinoma | CHOL | upregulated |
| Colon adenocarcinoma | COAD | ns |
| Esophageal carcinoma | ESCA | upregulated |
| Head and Neck squamous cell carcinoma | HNSC | ns |
| Kidney Chromophobe | KICH | downregulated |
| Kidney renal clear cell carcinoma | KIRC | upregulated |
| Kidney renal papillary cell carcinoma | KIRP | ns |
| Liver hepatocellular carcinoma | LIHC | upregulated |
| Lung adenocarcinoma | LUAD | ns |
| Lung squamous cell carcinoma | LUSC | ns |
| Pancreatic adenocarcinoma | PAAD | ns |
| Pheochromocytoma and Paraganglioma | PCPG | ns |
| Prostate adenocarcinoma | PRAD | ns |
| Rectum adenocarcinoma | READ | downregulated |
| Sarcoma | SARC | ns |
| Skin Cutaneous Melanoma | SKCM | ns |
| Stomach adenocarcinoma | STAD | upregulated |
| Thyroid carcinoma | THCA | ns |
| Thymoma | THYM | ns |
| Uterine Corpus Endometrial Carcinoma | UCEC | downregulated |

**Table S2. A series of PELI1-binding proteins supported by experimental evidence were obtained through STRING tool.**

| **Gene Symbol** | **Gene ID** |
| --- | --- |
| IRAK1 | ENSP00000358997 |
| IRAK4 | ENSP00000479889 |
| TRAF6 | ENSP00000433623 |
| RIPK1 | ENSP00000259808 |
| TRADD | ENSP00000341268 |
| RIPK3 | ENSP00000216274 |
| UBE2V1 | ENSP00000340305 |
| SMAD6 | ENSP00000288840 |
| UBE2N | ENSP00000316176 |
| TBK1 | ENSP00000329967 |
| PELI3 | ENSP00000322532 |
| PELI2 | ENSP00000267460 |
| TAB1 | ENSP00000216160 |
| IRAK2 | ENSP00000256458 |
| TRAF5 | ENSP00000261464 |
| TAB2 | ENSP00000356426 |
| TAB3 | ENSP00000368215 |
| TICAM1 | ENSP00000248244 |
| TICAM2 | ENSP00000415139 |
| TRAF2 | ENSP00000247668 |
| CHUK | ENSP00000359424 |
| RNF31 | ENSP00000315112 |
| CYLD | ENSP00000392025 |
| BIRC3 | ENSP00000263464 |
| BIRC2 | ENSP00000216160 |
| TIFA | ENSP00000354911 |
| TIRAP | ENSP00000376445 |
| SARM1 | ENSP00000468032 |
| TRAF3 | ENSP00000376500 |
| IKBKG | ENSP00000483825 |

**Table S3. The top 100 genes that correlated with PELI1 expression in LIHC tumor expression data of TCGA.**

**Table S4. Kinase, miRNA and transcription factor targets of PELI1 predicted by LinkedOmics.**

| **Enriched category** | **Gene Set** | **Leading Edge Number** | **NES** | **FDR** |
| --- | --- | --- | --- | --- |
| Kinase targets | Kinase_MAPK3 | 76 | 1.5858 | 0.080249 |
|  | Kinase_EGFR | 19 | 1.5746 | 0.084203 |
|  | Kinase_RAF1 | 8 | 1.5875 | 0.091682 |
|  | Kinase_CSNK1D | 11 | 1.5598 | 0.095307 |
|  | Kinase_PRKX | 26 | 1.5132 | 0.095812 |
| miRNA targets | GTTATAT,MIR-410 | 40 | 1.86 | <2.2e-16 |
|  | AAGGGAT,MIR-188 | 38 | 1.8355 | <2.2e-16 |
|  | ATAACCT,MIR-154 | 22 | 1.8048 | <2.2e-16 |
|  | ACACTAC,MIR-142-3P | 58 | 1.8041 | <2.2e-16 |
|  | AAGCACT,MIR-520F | 106 | 1.801 | <2.2e-16 |
| Transcription factor targets | TAANNYSGCG_UNKNOWN | 35 | 1.687 | 0.0050151 |
|  | V$EVI1_06 | 9 | 1.5812 | 0.0072947 |
|  | V$SMAD_Q6 | 83 | 1.5482 | 0.0074771 |
|  | V$E2F_Q4 | 86 | 1.5918 | 0.0075227 |
|  | V$ETS2_B | 124 | 1.5777 | 0.0075227 |

**Table S5. Differentially expressed genes between the high- and low-PELI1 expression groups.**
